# Temporal patterns of Amazonian insect acoustic activity

**DOI:** 10.1098/rstb.2024.0337

**Published:** 2025-06-12

**Authors:** Nia Howells, Alexander Charles Lees, Jos Barlow, Erika Berenguer, Liana Chesini Rossi, Jérôme Sueur, Martin J. Sullivan, Yan Gabriel Ramos, Oliver C. Metcalf

**Affiliations:** ^1^ Department of Natural Sciences, Manchester Metropolitan University, Manchester, M1 5GD, UK; ^2^ Lancaster Environment Centre, Lancaster University, Lancaster, Lancashire, UK; ^3^ Environmental Change Institute, Oxford University, Oxford, UK; ^4^ Jardim Botanico do Rio de Janeiro, Rio de Janeiro, Brazil; ^5^ Museum National d'Histoire Naturelle, Paris, Île-de-France, France; ^6^ Aiuká Consultoria em Soluções Ambientais, Forte Praia Grande, São Paulo CEP 11700-280, Brazil

**Keywords:** ecoacoustics, insects, Orthoptera, tropical forests, acoustic indices, passive acoustic monitoring

## Abstract

Insects are one of the most diverse taxa and are fundamental to the delivery of many ecosystem services. Despite their global ubiquity and ecological importance, there is little research on temporal variation in insect activity, especially in the tropics where the group is most diverse. Gaps in our knowledge of insects are compounded by a lack of robust methods to monitor their activity at fine timescales. Ecoacoustic techniques have emerged as an effective means to monitor a range of taxa over long periods. Here, we assess insect acoustic activity in the eastern Brazilian Amazon across daily and seasonal cycles over 2 years. We relate acoustic indices to two subsets of manually assessed activity—sonotype diversity and spectrogram coverage. We find evidence for daily and seasonal periodicity, with both the spectrogram coverage and number of insect sonotypes higher nocturnally. Insect acoustic activity peaks during the dry season. Of the five acoustic indices used, the Bioacoustic Index best predicted acoustic insect activity across both metrics. Our results indicate that passive acoustic monitoring can be an effective tool for assessing broad trends in insect phenology.

This article is part of the theme issue ‘Acoustic monitoring for tropical ecology and conservation’.

## 1. Introduction

Insects are taxonomically hyperdiverse [1,2], and play fundamental roles in delivering ecosystem services [3,4]. Despite their ecological importance, there is proportionately little research on insects [5]. Only around 20% of all insect species have been formally described, and of these [1], very little is often known about even the basic ecology of the majority—such as their trophic position, habitat preferences, phenological patterns and daily activity patterns. An understanding of these ecological patterns is fundamental to interpret complex population-level data [6]—and therefore in understanding the impacts of the entwined climate and biodiversity crises [7]. These knowledge gaps are especially problematic in tropical forests, which have received far less attention than habitats at temperate latitudes.

Passive acoustic monitoring (PAM) is increasingly used as an ecoacoustic approach to survey biodiversity, often in support of traditional field methods [8–10], and could be used to fill gaps in our knowledge of spatiotemporal trends in insect populations and community composition. Insect biophony dominates tropical soundscapes, with some other soniferous taxa actively avoiding competition with them in periods of peak acoustic activity [11–13]. Tropical forest insect biophony is primarily generated by katydids (Orthoptera: Tettigoniidae) and cicadas (Hemiptera: Cicadidae) producing broadband calls, and crickets (Orthoptera: Grylloidea) producing narrowband calls between 3 and 9 kHz. This leads to a high proportion of the acoustic energy in a soundscape being generated by insects, and the opportunity for effective ecoacoustic monitoring [14–17].

Ecoacoustic methods provide an opportunity to continuously monitor ecosystems over extended temporal periods [18] and can reveal the nature of spatiotemporal variation in insect biophony, potentially providing a basis for evaluating the biodiversity consequences of global environmental change. In tropical rainforests, PAM has recently started to be used to evaluate temporal variation in insect activity. A study from northern Amazonia showed that katydid and cricket activity peaked just prior to dawn [17], while another undertaken at Barro Colorado Island in Panama showed that katydid acoustic activity was associated with lunar light levels [19].

However, little research has been conducted on acoustic activity at seasonal scales, despite seasonality impacting insect biomass. With Amazonian dry seasons becoming longer and more intense owing to local land use and global climate changes [20,21], it is imperative that the biological consequences of these new trends on insect populations are better understood. Seasonality drives variation in resource availability, such that animals align their breeding activity to time periods when abiotic factors allow them to take advantage of periods of heightened ecosystem productivity [22–24]. There is now emerging evidence that climate change, especially increased variance, may be driving invertebrate declines [25,26] but lack of long-term monitoring, especially in the tropics limits our understanding of the magnitude of declines [27].

As with many acoustic datasets, and especially those intended for phenological studies at multi-year scales, the collection of a large volume of data presents an analytical challenge. Acoustic indices have been developed to rapidly interpret soundscapes based on their acoustic properties [28]—typically measuring variations in acoustic energy across time and frequency. These indices help to solve the problem of large datasets by summarizing and quantifying soundscapes and function at the community level instead of the population level [29,30]. Acoustic indices have been used to study insects in the neotropics and respond to insect sounds—showing daily and seasonal responses [31–33] and responses to changing habitats [17]. However, acoustic indices are not necessarily reliable in all contexts [34–36], and thus require manual validation.

Here, we explore whether PAM in tropical forests can provide evidence of insect acoustic phenology across daily and seasonal cycles in the eastern Brazilian Amazon. As insect acoustic activity has rarely been studied at seasonal scales in neotropical environments, we evaluate five acoustic indices to test whether they could predict two manual measures of insect acoustic activity: (i) the proportion of insect acoustic space use; and (ii) number of insect sonotypes per 15 s, to provide insight into differing aspects of acoustic phenology.

## 2. Methods

### (a) Study sites and acoustic data collection

Our study was conducted in the eastern Brazilian Amazon in the adjacent municipalities of Santarém, Belterra and Mojuí dos Campos (latitude −3.046, longitude −54.947) in Pará state. Our recordings were collected at the centre of three permanent 300 m forest transects maintained by the sustainable Amazon network [37]. The transects were located in *terra firme* forests in a region with an average temperature of 25°C, 1993 mm of annual average precipitation and five months dry season (months with <100 mm rainfall) typically lasting between July and November [38].

To record forest biophony, we used Frontier Labs bioacoustic recording units [39] with a 16 bit sampling rate of 44.1 kHz. Recorders were placed 7–10 m high with the microphone in a downward-facing position and 10–20 m perpendicular to the transect. We placed recorders away from directly overhanging vegetation to limit geophony from leaves and branches and to avoid sound obstruction. We set the microphones with a fixed gain pre-amp of 20 dB, an 80 dB signal-to-noise ratio and 14 dBA self-noise, an 80 Hz high-pass filter to filter out low-frequency wind noise and a flat frequency response (±2 dB) from 80 Hz to 20 kHz. We programmed them to record 1 min every 10 min (1 min on, 9 min off) in .wav format between 1 January 2018 and 31 December 2019. Owing to technical failure, 77 066 files were missing. This led to a total of 238 294 files for 3972 h of recordings, with recording completeness between 73% and 78% across the three locations.

### (b) Analysis

We evaluated five acoustic indices to test whether they could predict two manual measures of insect acoustic activity: (i) the proportion of insect spectrogram coverage; and (ii) number of insect sonotypes per 15 s. We derived these two features from a manual assessment of a subset of the audio dataset, later smoothed through generalized linear mixed models (GLMMs). Next, as our audio dataset contained temporal gaps owing to equipment failure and extended periods of rainfall, we used generalized additive models (GAMs) to predict acoustic index values across the daily and annual cycles. Finally, using the index values predicted by the general additive models as input, we used the GLMMs to predict insect activity across the daily and annual cycles.

#### (i) Insect acoustic activity data

To validate the relationship between acoustic index values and insect acoustic activity, we manually estimated insect activity within the 3−10 kHz frequency band over a subset of the dataset. Firstly, we visually inspected the spectrogram and estimated the percentage area to the nearest 5% within the 3−10 kHz range that contained insect-related sounds (labelling by N.H.)—hereafter named ‘spectrogram coverage’. We assessed what sounds were coming from insects by inspecting spectrograms in Raven Pro v. 1.5 [40] and by listening wherever necessary. We used random sampling stratified by time of day, month, year and recording location to select 1137 files. After manually removing files containing rain, 890 were suitable for manual annotation. To reduce the time needed for manual validation, we took estimations using the first 15 s of each file as they were easier to visually assess and an initial qualitative assessment indicated there was little substantial change in insect activity between the first 15 s of a file and the following 45 s. We used reference libraries of identified sound files from the region to help with this process, primarily derived from three online sound libraries: Xeno-Canto, Macaulay Library and AmphibiaWeb [41–43].

Secondly, we counted the number of unique insect sounds, or sonotypes, present in each 15 s recording (labelling by N.H. and O.C.M.), using the same subset of data. After the first 70 files, it became apparent that this process took significantly longer, and thereafter we decided to only use every other file in the spectrogram coverage dataset, totalling 480 files. Although this led to an imbalance in sample stratification, we chose to retain all labelled data as we believe the modelling process has adequately handled the data imbalance. Counting of insect sonotypes was conducted using Raven Pro, where each sound file was labelled with the number of visually unique sonotypes identified within that recording.

#### (ii) Calculating acoustic indices

**Figure 1.**
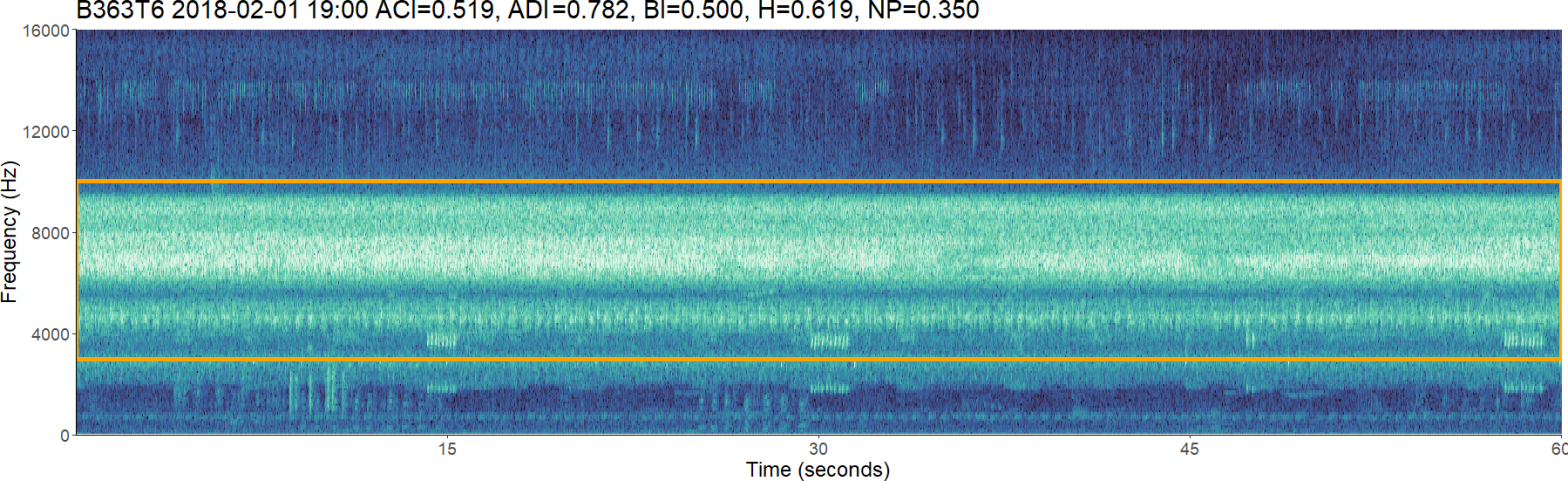
Spectrogram showing the insect sound activity between the chosen 3 and 10 kHz range (orange lines) from which we took all manual and index measurements. Scaled acoustic index values are shown in the plot title. This file corresponds to a 1 min recording on 1 February 2018 at 19.00. This was selected as the ACI value equals the third quartile value for all acoustic complexity index scores. ACI, acoustic complexity index; ADI, acoustic diversity index; BI, biacoustic index; H, acoustic entropy index; NP, number of frequency peaks.

We calculated five acoustic indices in the R statistical program (v. 4.2.3) [44] using the packages ‘soundecology’ [45] and ‘seewave’ [46]. Smaller, ecologically appropriate frequency bandwidths have been shown to reduce the effects of masking from non-target taxa [47]. Consequently, we chose a frequency bandwidth of 3–10 kHz (figure 1), based on scanning the acoustic files and previous studies that show this frequency range to be heavily used by soniferous insects, especially crickets [14,17,31]. We used *ffilter* from ‘seewave’ to apply these frequency ranges where minimum and maximum frequency limits were not included for index function arguments (table 1). We calculated all indices for the 1 min recordings and scaled them to between 0.0001 and 0.9999 using the scales package [53].

**Table 1.** A list of the acoustic indices employed, their description and function.

index	description (following Bradfer-Lawrence *et al*. [48])	function used (values in Hz) all parameters not given are default
acoustic complexity index (ACI [49])	a measure of sound complexity, computed as the amplitude difference between successive Fourier windows for each frequency band	soundecology:: acoustic_complexity (min_freq = 3000, max_freq = 10 000)
acoustic diversity index (ADI [50])	high values show even amplitude across frequency bands	(1) frequency filter seewave:: ffilter(from = 3000, to = 10 000) (2) soundecology:: acoustic_diversity()
bioacoustic index (BI [51])	a product of amplitude—relative to the quietest frequency band—and the number of frequency bands. Higher values imply a greater disparity between the loudest and quietest bands	soundecology:: bioacoustic_index(min_freq = 3000, max_freq = 10 000)
acoustic entropy index (H [29])	a product of spectral (frequency) and temporal entropy	(1) frequency filter seewave:: ffilter(from = 3000, to = 10 000) (2) seewave:: H()
number of frequency peaks (NP [52])	the number of frequency peaks on the mean frequency spectrum (i.e. the mean relative amplitude of the frequency distribution)	1.seewave:: meanspec(correction=”amplitude’) (1) filter mean spectrum to frequencies >300 (2) seewave::fpeaks() (3) take sum of peaks in frequencies >3000 and <10 000

The calculation of acoustic indices is influenced by geophony [54] and recorder malfunction, which could give spurious results and lead to incorrect inferences. We undertook a multistep process to remove these files from the overall dataset. First recording periods containing heavy rainfall were screened out using the hardRain package [55]. Next, a sample of 1000 sound files were visually scanned and labelled as ‘problematic’ where there were obvious problems, ‘intermediate’ where there were some problems such as light rain but biotic sound was still clearly visible, and ‘normal’ where no problems were noted. Mahalanobis distances were calculated using all index scores for the entire files. Mahalanobis distances over 12 were found to contain the majority of problematic sound files (electronic supplementary material, appendix 1 figure S1) and were removed from analysis. Time periods where there were clear problems with the microphone were found through visually inspecting daily patterns in index scores split by month, transect and year (electronic supplementary material, appendix 1 figure S2). After data cleaning methods, 207 812 1 min files (87%) of files were retained.

#### (iii) Modelling insect acoustic activity

We modelled the manual values of insect activity against the acoustic index values using GLMMs with the GlmmTMB package [56]. We built separate models for each metric of insect activity but applied the same modelling approach to both. First, we built a global model, modelling the manual measure of insect activity against each of the five acoustic indices as linear and quadratic terms, plus transect name and year as fixed effects. For the proportion of spectrogram coverage model, we used a beta distribution with a logit link, and for the sonotype model, we used a Poisson distribution. We then used the dredge function in the MuMIn R package [57] to identify the best models using Akaike information criteria for small sample size (AICc) scores [58] and built an average model using all models with delta AICc values of less than 2 from the best model (see the electronic supplementary material, appendix 2, for model summaries and diagnostics). We assessed model performance through fivefold cross-validation of the best-performing model for each metric, repeated 100 times and calculated the mean and standard deviation of root mean squared error (RMSE) and mean absolute error (MAE) for both models. As some data was missing across the annual cycle owing to recorder failure, intense periods of rainfall and the data cleaning process, we used GAMs in the mgcv package [59] to model each acoustic index over 24 h and 12 months cycles. We used separate models for each index, with smooth terms for hour as a numeric value between 1 and 24, and day of the year as a numeric value between 1 and 365, with cyclic cubic regression splines, four basis functions, an interaction term between transect name, and year of recording. We used a Gaussian distribution in every model. We chose a low number of basis functions to allow the model to better reflect general seasonal trends rather than daily variation.

Finally, we used the predicted acoustic index values across the daily and annual cycles as input values for the GLMMs to predict insect acoustic activity over these cycles. To propagate uncertainty from both models we (i) randomly drew values for each index from a normal distribution where the mean was the value of the index predicted by the GAM and the standard deviation was the standard error of the GAM model estimate; (ii) used the predicted index values as input for the GLMM prediction; and (iii) randomly drew a predicted insect activity value from a normal distribution where the mean is the GLMM prediction, and the standard deviation is the GLMM prediction standard error. This process was iterated 1000 times, producing a distribution of predicted insect activity values.

## 3. Results

### (a) Insect acoustic phenology

**Figure 2.**
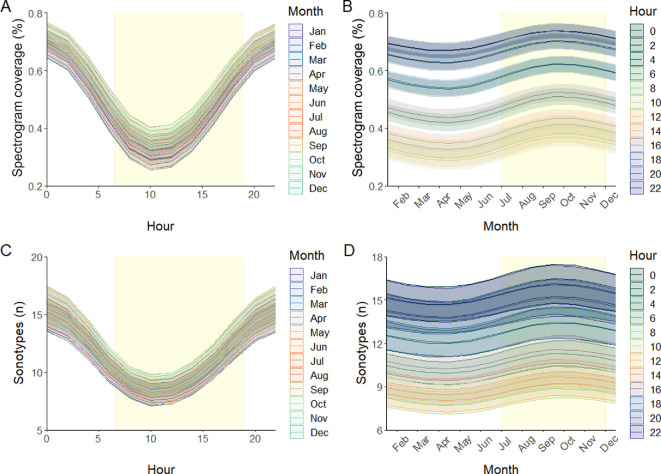
Insect acoustic activity over daily and annual cycles. Panels A and B show predictions of the spectrogram coverage by insects, while panels C and D show the number of insect sonotypes per 15 s. The rectangles in panels A and C show the period between sunrise and sunset on 1 January 2019, while the rectangles in panels B and D show the typical dry season.

We found that Amazonian insect activity had strong periodicity, showing both seasonal and daily cycles. This was reflected in both our metrics of insect activity, with similar patterns across the proportion of spectrogram coverage and number of sonotypes per 15 s file (figure 2). Boxplots of the manually assessed data suggest that trends were similar across the three sites (electronic supplementary material, appendix 3 figure S1). The daily cycle peaked between 20.00 and 05.00 for both metrics, with lower diurnal values between 07.00 and 15.00 (figure 2A,C). The maximum manually assessed spectrogram coverage was at 21.00 with a mean of 0.86 ± 0.06 s.d. and lowest at 08.00 with a mean of 0.30 ± 0.17, a difference of 0.56, larger than both the RMSE (0.18 ± 0.01) and MAE (0.14 ± 0.01) for the model. The maximum number of manually assessed sonotypes per 15 s occurred at 23.00 (17.85 ± 7.40 s.d.), with the lowest at 12.00 (5.82 ± 2.58 sonotypes), with the difference of 12.03 again exceeding the estimated model error rates (RMSE = 4.28 ± 0.37, MAE = 3.25 ± 0.27). Daily patterns were similar throughout the year.

Models showed peak insect acoustic activity from July to November with maximum values recorded in September, corresponding to the height of the dry season in the region (figure 2B,D), with the lowest values in April and May. Differences were not as pronounced between seasons as between times of day, with peak manually estimated values of spectrogram coverage at its maximum in September, the dry season, with a mean of 0.67 ± 0.23 s.d., and at its minimum in March, the wet season, with values of 0.44 ± 0.32, although the differences between the mean maximum and minimum values did exceed the estimated model error. Again, sonotypes per 15 s showed similar trends with the highest manually detected mean sonotypes in July (13.2 ± 5.45) and lowest in March (9.38 ± 4.92), however in this case the difference between mean maximum and minimum values, 3.81, was lower than the model RMSE (4.28 ± 0.37) but larger than MAE (3.25 ± 0.27). Seasonal patterns were similar across the daily cycle, but for spectrogram coverage the difference during different parts of the daily cycle was clearly visible, with much lower values at midday, followed by dawn, the middle of the night, then highest values at dusk (figure 2B).

### (b) Acoustic indices as predictors of acoustic activity

Our GLMMs uncovered different relationships between each of the indices and the manual metrics, but also revealed that each index varied in its response to each manual metric (figure 2A,B). Biacoustic index (BI), acoustic entropy index (H) and number of frequency peaks (NP) showed relatively similar responses to both proportion of spectrogram coverage and sonotypes per 15 s, but acoustic complexity index (ACI) and acoustic diversity index (ADI) showed divergent responses between the manually measured metrics. Interestingly, ACI and BI showed divergent responses to the proportion of spectrogram coverage. ACI had a strong negative relationship and BI showed a curvilinear positive relationship, they both however showed similar responses to variation in sonotypes per 15 s, with both values increasing initially with sonotypes, before starting to rapidly decline around 12 sonotypes per 15 s.

**Figure 3.**
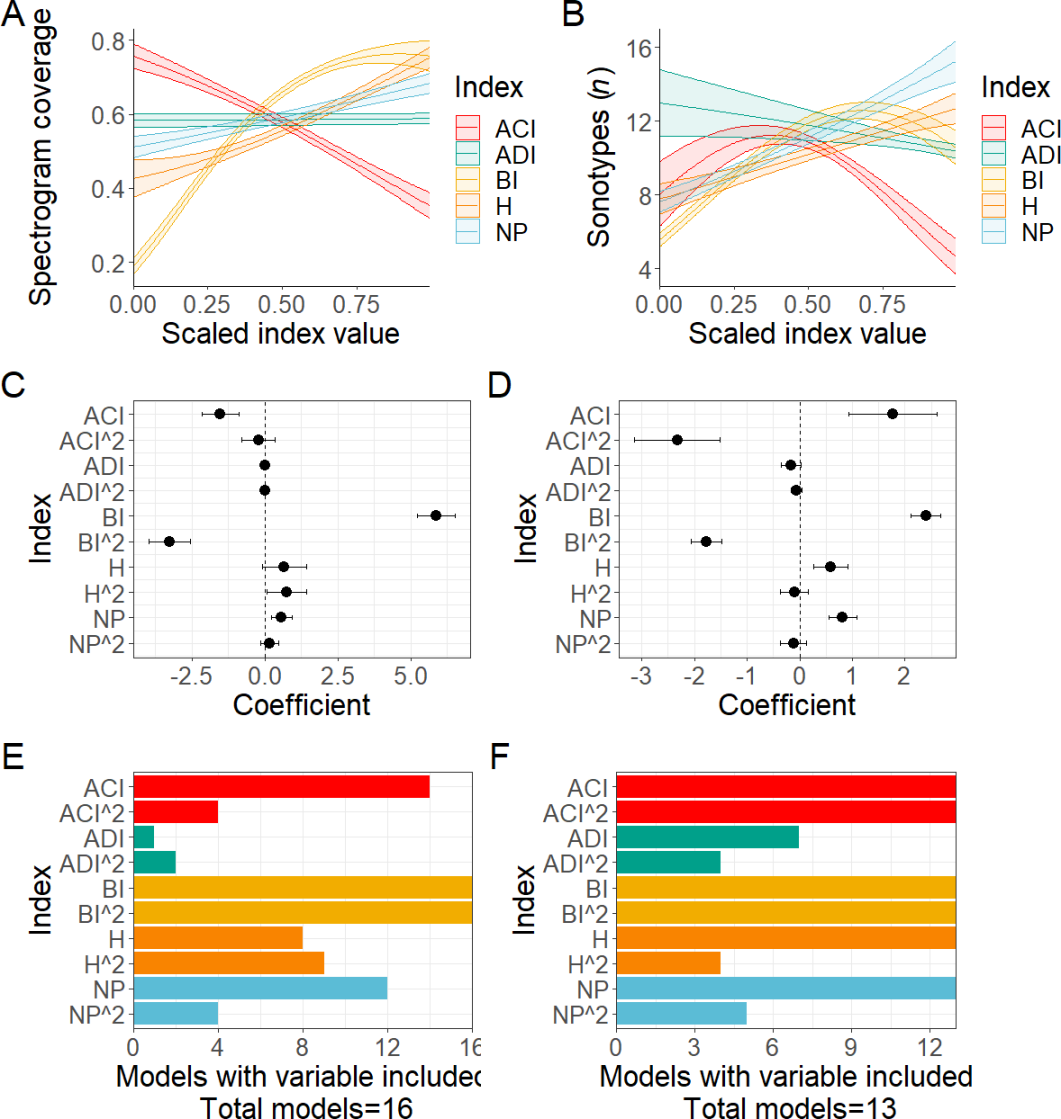
The relationship of five acoustic indices to (A) proportion of insect spectrogram coverage (%) and (B) number of insect sonotypes per 15 s file, respectively. The coefficient effect size of the relationship with insect spectrogram coverage (C) and number of insect sonotypes (D) for the five indices. The number of times each index term was included in the models selected for inclusion on the averaged models used for (E) proportion of acoustic space and (F) sonotypes models. (E) has a total of 16 selected models and (F) a total of 13. ACI, acoustic complexity index; ADI, acoustic diversity index; BI, biacoustic index; H, acoustic entropy index; NP, number of frequency peaks.

Both GLMM models showed that BI was a strong predictor of insect acoustic activity (figure 3C,D), with BI being included in all selected models for both manual metrics (figure 3E,F). For spectrogram coverage the linear term for ACI and NP, and the quadratic term for H were also strong predictors and included in the majority of selected models, while for sonotypes per 15 s the quadratic term for ACI and the linear terms for H and NP were strong predictors and included in all selected models, with the linear term for ADI also included in most selected models.

**Figure 4.**
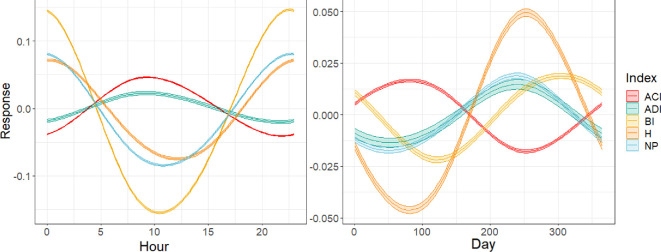
Generalized additive model results showing response in acoustic index values to the daily and annual cycles. Colour fills indicate 95% confidence intervals of the models. ACI, acoustic complexity index, ADI, acoustic diversity index; BI, biacoustic index; H, acoustic entropy index; NP, number of frequency peaks.

Our GAMs found similar periodicity over the daily cycle across all indices, although the direction of the trend was reversed for ACI and ADI, which showed peaks in values around 12.00, compared to BI, H and NP which showed their lowest values around 12.00 (figure 4). BI exhibited the strongest daily cycle. By contrast, there was variation in the trends across the annual cycle, with ACI, highest around March and lowest around August, BI peaking in October and lowest in April and H the inverse of ACI but more pronounced, although ADI and NP were very similar with the highest values in July and lowest in February.

## 4. Discussion

### (a) Insect acoustic phenology

Using PAM, we were able to illustrate phenological patterns in insect acoustic activity across the daily and annual cycles. The daily cycle showed, as expected, more insect acoustic activity during the night and less during the day, a common feature of tropical soundscapes where Orthoptera typically produce constant sustained acoustic signals at night [60]. This matches the findings of previous PAM studies in the neotropics [17,32], but also a range of studies looking at soundscapes more broadly [61,62]—that insects dominate tropical soundscapes. However, in contrast to previous PAM studies, we studied acoustic activity across all insect sonification between 3 and 10 kHz and across the whole 24 h cycle. This allowed us some strong, broad insights, although the scale of the analysis undertaken here may have limited the detail of the analysis available, and may explain why the nocturnal activity levels in our study peaked around 00.00 as opposed to just before dawn [17] or in dual peaks [19]. Similarly, other studies have found varying daily patterns across the neotropics; Sueur [63] found pronounced dawn and dusk choruses by cicadas in tropical rainforests in Mexico, and Gomez-Morales & Acevedo-Charry [64] found a dusk chorus of insects in sub-Andean forests in Colombia, while our study did not find such chorusing. This apparent disparity in results highlights the importance of understanding the factors underpinning temporal structuring of soundscapes, and further research is required to assess whether these apparently contradictory results pertain to methodologies and analytical approaches, or to geographical variation in soundscape phenology.

Seasonal trends exhibited increased activity between July and November, aligning with the dry season rather than the wet season. Although the seasonal temporal variation was less marked than in the daily cycle, the timing of this seasonality supports a more direct relationship with ecosystem productivity, which follows the same seasonal cycle with the leaf area index peaking in October and at a minimum in July [65]. Gomez-Morales & Acevedo-Charry [64] also found reduced insect activity during periods of higher rainfall, which they speculated was behaviour intended to reduce masking, although their study was restricted to rainy periods and did not look at seasonal effects. Additionally, Oliveira *et al*. [32] found stark differences between daily cycles in the wet and dry seasons in seasonally dry tropical forest in Brazil with night-time peaks only in the wet season. By contrast, we found that the daily pattern was not impacted by seasonality. It remains to be seen if the patterns we found in eastern Amazonia, which exhibits clear rainy and dry seasons, reflect other forests across the basin, such as the aseasonal regions in the northwest or highly seasonal transitional regions in southern Amazonia.

The proportion of insect spectrogram coverage and the number of insect sonotypes per 15 s showed remarkably similar daily and seasonal trends, suggesting that factors temporally structuring soundscapes impact both the abundance and species richness of sonifying insects. However, other studies [19,64] have used classification methods to identify variation in phenology at much finer taxonomic granularity, suggesting that our results, while reflecting broad trends, probably mask huge complexity in insect acoustic activity phenology, even in the region. PAM remains a valuable tool to study this, but gaining better libraries of regional insect sonotypes can aid in developing effective species-level classifiers, as demonstrated with other taxonomic groups [65], to resolve this complexity. Furthermore, it is not clear whether acoustic activity provides a good proxy for abundance, a metric that is used far more widely to understand insect seasonality [66,67], and more studies linking traditional metrics of insect abundance to soundscape metrics would be valuable.

### (b) Ecoacoustic methods

We found that PAM, coupled with ecoacoustic analysis techniques, was an effective method for revealing the phenology of soniferous insects. However, we revealed strong variation in acoustic index responses to the manually measured metrics of insect biophony, reflected in the relatively high estimated error rates for our predictions. While the predictive ability of the GLMM models remained robust enough to maintain confidence in the trends reported here, this relatively high error probably reflects the limitations of acoustic indices in being unable to fully remove the influence of non-insect sounds, even when calculated at optimal frequencies. In addition, the curvilinear response of some of the indices implied that there was a degree of saturation occurring in responses, for instance, the BI response to spectrogram coverage, or even that indices were perhaps unable to differentiate between a very full soundscape and a very quiet one (BI and ACI responses to number of sonotypes per 15 s). This highlights the benefits of using a suite of acoustic indices simultaneously [47], and the necessity of using appropriate statistical methods for multivariate analysis.

Of the five acoustic indices used, the BI best predicted acoustic insect activity across both metrics. This may be because this index is sensitive to the distribution of acoustic energy across frequency bands, and insect calls typically cover entire frequency bands, consistently and loudly, across a spectrogram. It also corresponds with the way spectrogram coverage was estimated based on the amount of space used on the spectrogram. However, other studies have found that BI was not correlated with insect sound [31] and their sites showed strikingly different daily cycles by index. This variation, alongside the differing responses of indices to the different metrics of insect acoustic activity in our own study, further highlights the lack of generalizability in acoustic index response to soundscapes [34,35]. This emphasizes the need to interrogate the local ecological causes of index responses by doing at least some manual listening to gather context-specific biodiversity correlates.

This study was necessarily limited by several factors, for example, precise rainfall data were not available to include in our models, which would have helped elucidate the relationship between insect acoustic activity and climate or weather effects. Improving meteorological data will allow improved studies in the future. The small number of stationary recorders restricted our ability to investigate the differences between understory and canopy communities, as well as effects of habitat type. Our study was limited to 2 years, but longer-term datasets will permit the impacts of stressors such as El Niño cycles to be measured. The frequency range in this study was limited to 24 kHz, meaning that insects calling at ultrasonic frequencies were not measured. As costs for acquiring, storing and analysing data decrease, collecting broader-frequency and longer-term datasets will become more feasible, overcoming these limitations.

## 5. Conclusion

Despite their importance, there is little research on temporal variation in insect activity, and we lack robust methods to monitor their activity at fine timescales. Our study using PAM techniques to monitor insect acoustic phenology in the Amazon showed a clear daily cycle and a seasonal pattern in acoustic activity following ecosystem productivity, with the peak during the dry season—providing baseline data for insect acoustic activity in this region. Future efforts need to be directed towards building more comprehensive sound libraries of insect biophony to determine acoustic phenology for individual species. Additionally, modelling acoustic data against meteorological data, such as rainfall and humidity, would be valuable given the unknown effects of climate change on insect activity. PAM techniques can provide much-needed insight into the behaviour of highly biodiverse groups, such as insects, by enabling continuous, non-invasive monitoring across large spatial and temporal scales, offering new opportunities to study their ecological roles and responses to environmental changes. We conclude that PAM can be an effective tool for assessing broad trends in insect phenology.

## Data Availability

The data and code can be found on Zenodo [68]. Supplementary material is available online [69].
